# Prevalence of Hepatitis D in People Living with HIV: A National Cross-Sectional Pilot Study

**DOI:** 10.3390/v16071044

**Published:** 2024-06-28

**Authors:** Georgios Schinas, Nikolina Antonopoulou, Sofia Vamvakopoulou, Olga Tsachouridou, Konstantinos Protopapas, Vasileios Petrakis, Emmanouil C. Petrakis, Despoina Papageorgiou, Simeon Metallidis, Antonios Papadopoulos, Emmanouil Barbounakis, Diamantis Kofteridis, Periklis Panagopoulos, Alexandra Lekkou, Fotini Paliogianni, Karolina Akinosoglou

**Affiliations:** 1 School of Medicine, University of Patras, 26504 Patras, Greece; georg.schinas@gmail.com (G.S.); nikolina.antonopoulou@gmail.com (N.A.); dspn.pap96@gmail.com (D.P.); 2 Department of Microbiology, University General Hospital of Patras, 26504 Patras, Greecefpal@upatras.gr (F.P.); 3 Departments of Internal Medicine and Infectious Diseases, University General Hospital of Thessaloniki “AHEPA”, 54636 Thessaloniki, Greece; olgatsachouridou.iasis@gmail.com (O.T.); symeonam@auth.gr (S.M.); 4 4th Department of Internal Medicine, Medical School, National and Kapodistrian University of Athens, Attikon University General Hospital, 12462 Athens, Greece; kprotopapas@hotmail.com (K.P.); antipapa@med.uoa.gr (A.P.); 5 Departments of Internal Medicine and Infectious Diseases, Democritus University of Thrace, 68100 Alexandroupolis, Greece; vasilispetrakis1994@gmail.com (V.P.); ppanago@med.duth.gr (P.P.); 6 Departments of Internal Medicine and Infectious Diseases, University General Hospital of Heraklion, 71500 Heraklion, Greece; mpetrakis86@gmail.com (E.C.P.); barbouman2003@yahoo.gr (E.B.); kofterid@uoc.gr (D.K.); 7 Departments of Internal Medicine and Infectious Diseases, University General Hospital of Patras, 26504 Patras, Greece; alekkou@yahoo.gr

**Keywords:** hepatitis D, HIV infections, hepatitis B, coinfection, Greece, epidemiological monitoring

## Abstract

This study assesses the prevalence of hepatitis D virus (HDV) in people living with HIV (PLWHIV) in Greece. Given the compounding effects of HDV and hepatitis B (HBV) on liver disease progression, as well as the emergence of new therapeutic options such as bulevirtide, understanding regional disparities and the epidemiological impact of such co-infections is vital. A cross-sectional analysis was conducted utilizing 696 serum samples from PLWHIV attending five major university hospitals. The methodology included HDV antibody detection by ELISA and HDV RNA confirmation. Of the 30 HBsAg-positive samples analyzed, the study population was primarily male (93%), with a median age of 54 years. Participants had been on antiretroviral therapy for a median of 10 years, and the median CD4 count was 738 (539–1006) copies/mL. Additional serological findings revealed a 7% prevalence of hepatitis C virus (HCV) IgG antibodies and a 55% prevalence of hepatitis A virus (HAV) IgG antibodies. Seroreactivity for syphilis (RPR/VDRL/TPHA positive) was identified in 33% of the participants. The results indicated a low HDV prevalence, with only one individual (3%) testing positive for anti-HDV IgG antibodies and none for HDV RNA. This indicates a lower prevalence of HDV among PLWHIV with chronic HBV in Greece compared to global data.

## 1. Introduction

The hepatitis D virus (HDV) is an RNA virus that requires the biological mechanisms of the hepatitis B virus (HBV) to complete its life cycle within the hepatocyte, thus earning it the title of a “satellite” virus [1]. HDV hepatis may, therefore, occur either as an acute co-infection with HBV or as a superinfection in a chronic HBV carrier [2]. Although a 95% virological cure rate is observed in individuals after acute HDV/HBV co-infection, the outcomes differ significantly for HDV superinfections, with over 90% of the cases progressing to chronic co-infection [3]. In fact, HBV/HDV infection is associated an increased risk of poor long-term outcomes (cirrhosis, HCC and death) compared to HBV monoinfection. Compared with HBV monoinfection, the risk of progression to most phases of severe disease is at least doubled, with the risk of cirrhosis and HCC being substantially higher than for HBV [4,5]. Of note, HDV co-existence has been described as the strongest predictor of adverse outcomes in HBV patients, associated with a 6-fold higher risk of cirrhosis [6]. In line with this, it seems that older age, male sex, elevated liver enzymes, diabetes mellitus, obesity and co-infection with HCV influence the outcome of HDV infection and disease [7].

An estimated 62 to 72 million people worldwide are affected by hepatitis D [8]. While the prevalence of HDV among HBV/HIV co-infected individuals is less well-established, the potential for HDV co-infection in people living with HIV (PLWHIV) is recognized, particularly due to the overlapping transmission routes shared among hepatotropic viruses and HIV [9,10]. Additionally, the chronic immunocompromised state of these individuals naturally predisposes them to superinfection, particularly among those who are HBsAg positive. The risk becomes evident when taking into account the increased global prevalence of HBV in PLWHIV, calculated at 7.4% [11]. This coexistence has the potential to complicate the management of the three diseases and may exert a direct influence on their respective outcomes, particularly by accelerating liver disease progression. HDV infection, in particular, has been linked to an increased risk of hepatitis flares, liver cirrhosis, hepatic decompensation, and death in patients with concurrent HIV/HBV infection [12].

Moreover, the intersection of HDV and HIV infection presents a public health concern, particularly due to the hematogenous transmission potential of both viruses. This concern is underscored by findings from a recent, comprehensive pan-European study [13]. In this study, which evaluated a large cohort of PLWHIV, it was found that the prevalence of HDV co-infection was exceedingly high, at 50.5% (95% CI: 45.3–55.7%), among intravenous drug users, a subgroup in which 65% exhibited active HDV replication. Conversely, PLWHIV who do not use intravenous drugs showed a prevalence of HDV infection akin to the general population, at 4.7% (95% CI: 3.5–5.9%). In Southern Europe, in particular, the prevalence of HDV infection among non-users was significantly higher, recorded at 8.1% (95% CI 1.3–14.8%), indicating regional variations.

Unveiling regional disparities and pinpointing the epidemiological impact of HDV/HBV co-infection in PLWHIV is of imminent importance. This is particularly pertinent given the recent approval of bulevirtide for the treatment of chronic HDV infection, a first-of-its-kind medication targeting HDV. In the context of this significant advancement in HDV therapeutics and the dynamic progression of HIV clinical management, our study aims to investigate the prevalence of HDV in PLWHIV in Greece, a country where specific data on this topic remain absent.

## 2. Materials and Methods

### 2.1. Study Design

This cross-sectional study was conducted to assess the prevalence of hepatitis D virus (HDV) among people living with HIV (PLWHIV) utilizing a biobank assembled from 696 serum samples collected from individuals attending the outpatient HIV clinics of 5 major university hospitals/HIV referral centers in Greece, including the University General Hospital of Patras, University General Hospital of Athens “Attikon”, University General Hospital of Thessaloniki “AHEPA”, University General Hospital of Herakleion and University General Hospital of Alexandroupolis, as per the SHIP study previously described [14]. The current study was approved by the Research Ethics Committee and Institutional Review Board (IRB) of all the participating institutions and was led by the University General Hospital of Patras and the respective university department (#147/13-03/2024 and 16465/04-04-2024). All the participants provided informed consent for the use of their biobank samples in this research. This study was conducted in accordance with the ethical standards of the Declaration of Helsinki.

### 2.2. Participants

Participants were selected based on their enrollment in the biobank, as previously described [14], and confirmed HIV-positive status. Patients were included in this study, in the presence of co-infection with hepatitis B virus (HBV), as determined by the presence of the hepatitis B surface antigen (HBsAg). Of the 696 serum samples collected from PLWHIV, 30 subjects were positive for HBsAg. Examination of these patients’ records also revealed that past HBV-DNA testing was negative for active viral replication. Following the provision of signed informed consent for the use of their samples in this study, patients were included for subsequent HDV testing.

### 2.3. Laboratory Procedures

Serum samples from HBsAg-positive PLWHIV were screened for HDV infection. The detection of HDV was performed in two stages:

Anti-HDV Antibody Testing: An initial enzyme-linked immunosorbent assay (ELISA) was utilized to test for the presence of anti-HDV antibodies (IgG and IgM), specifically the LIAISON^®^ XL murex Anti-HDV by DIASORIN Biotechnology, Sallugia, Italy.

HDV RNA Testing: Samples that tested positive for anti-HDV antibodies underwent further testing for HDV RNA to confirm active HDV infection. The process involved two main steps: (A) RNA isolation using the QIAamp Viral RNA kit by Qiagen, Hilden, Germany and (B) reverse transcription, amplification, and detection utilizing the genesig Hepatitis Delta Virus Kit from PrimerDesign, Southampton, UK along with the RotorGene 5Plex from Qiagen. The standardization of the procedure was performed using the Paul-Ehrlich-Institut (7657/12) International Standard for HDV RNA.

Laboratory tests were conducted at the Department of Microbiology at the University General Hospital of Patras, adhering to the manufacturer’s instructions and standard operating procedures for each assay.

### 2.4. Data Collection and Statistical Analysis

Data on the participants’ demographics, HIV-related clinical parameters, liver enzyme tests and results of HDV testing were collected from the patient records and anonymized for analysis. Given the exploratory nature of the study and the small number of anti-HDV-positive cases, the analysis focused on the calculation of the HDV prevalence among HBsAg-positive individuals. Descriptive statistics were used to summarize the data utilizing SPSS software (version 27.0, IBM Corp, Armonk, NY, USA).

## 3. Results

In total, 30 PLWHIV were included in this study. In this subset of PLWHIV, all the individuals were on antiretroviral therapy, with a universally undetectable viral load (Table 1). The median age of the participants was 54 years, and a significant majority were male (93%). Among the risk factors, men who have sex with men (MSM) were prevalent, accounting for 50% of the group. The modes of HIV transmission predominantly involved sexual contact (60%), with a considerable proportion being unaccounted for or unknown (37%).

The HIV stage distribution indicated that the majority (73%) were asymptomatic (A1–A3), while those with clinical manifestations either non- or indicative of AIDS (B1–B3 and C1–C3) constituted 17% and 10%, respectively. The treatment landscape was dominated by NRTI-based regimens (90%), in combination with INSTI-based therapies (71%). Patients not receiving NRTI (tenofovir alafenamide (TAF) or tenofovir disoproxil fumarate (TDF))—with the expected concomitant activity against HBV—as part of their ART regimen were contraindicated to receive TDF or TAF due to resistance or intolerance issues; hence, they received entecavir (as an HBV antiviral agent, not as part of their ART regimen). NNRTIs and PIs were part of the treatment for 21% and 30% of the cohort, respectively.

This study revealed a low prevalence of anti-HDV, with only one individual (3%) testing positive for anti-HDV IgG among the thirty HBsAg-positive participants (Table 2). This individual was a 23-year-old male, newly diagnosed with HIV (1-year post-diagnosis), and identified as MSM. His HIV stage was A2, and he was on antiretroviral therapy with TDF, 3TC and darunavir/ritonavir (DRV/r). Laboratory evaluations showed a white blood cell count of 3.57 × 10^3^/µL, platelet count of 183 × 10^3^/µL, and liver enzymes (SGOT and SGPT) of 55 U/L each. His albumin and globulin levels were 4.71 g/dL and 14.8 g/dL, respectively. The HIV viral load was undetectable, and the CD4 count was 406 cells/µL. Additionally, this individual tested positive for HAV antibodies and RPR/VDRL/TPHA. Further HDV-RNA testing proved negative, indicating the absence of active HDV infection in this individual. The assessment of the other infectious diseases in the population of HbsAg (+) PLWHIV indicated a notable presence of HAV IgG antibodies (55%) and a significant rate of syphilis seroreactivity (33%). Other concurrent infections, including HCV (7%), TB (4%), and other STDs (4% for unspecified, 11% for gonococcal disease, and 7% for chlamydia), were less frequently observed.

Laboratory evaluations showed median WBC and PLT counts within normal ranges, suggesting no significant hematological abnormalities (Table 2). The liver enzymes (SGOT and SGPT) and markers of renal function (Ur and Cr) were within normal or near-normal ranges, underscoring a generally stable physiological state in the cohort.

## 4. Discussion

This is the first study to explore HDV seropositivity among HbsAg-positive PLWHIV in Greece. Our results indicate a relatively low prevalence of HDV co-infection, with 3% of the participants testing positive for anti-HDV IgG antibodies and none for HDV RNA, within a representative cohort of 30 HBV/HIV co-infected patients in Greece.

Epidemiologic studies have shown that the prevalence of HDV/HBV co-infection in PLWHIV varies globally, influenced by geographical, socio-economic, and demographic factors [15]. For instance, in our neighboring country of Italy, a recently published prospective cohort consisting of 316 PLWHIV identified the presence of anti-HDV antibodies in 15.2% of all the participants [16]. Subsequent HDV-RNA testing confirmed 17% of individuals with positive anti-HDV antibodies to have an active HDV infection, and among this group, a significant 71% displayed indications of advanced liver disease. Similarly, the ICONA study, including HsAg(+) PLWHIV, showed an anti-HDV (+) rate of 18.8%, 66.3% of which were positive for HDV RNA [17]. The prevalence declined over the observation period, while the HDV patients were more often males, intravenous drug users and/or HCV co-infected. The authors underlined the fact that HDV infection among PLWH is underdiagnosed, although HDV entails a high risk of liver disease progression [17]. The Dat’AIDS Cohort examined 2406 HBsAg+ PLWHIV and showed a 15.6% anti-HDV+ overall, the majority of 56.5% anti-HDV+ in IVDU, 38.2% anti-HDV+ in patients from Eastern Europe and 42.4% anti-HDV+ in HCV/HBV co-infected patients [18]. On the other hand, the ATHENA cohort in the Netherlands found just a 7.1% prevalence of anti-HDV among PLWHIV [19]. In line with their findings, another recent study from the United States placed the prevalence of HDV co-infection among PLWHIV at 4.0%, among whom 41.7% had detectable HDV RNA [20].

Overall, the substantial heterogeneity in terms of patient populations and study designs precludes confident interpretation of the prevalence of HDV/HBV co-infection. Furthermore, the predictive value of diagnostic assays for HDV has evolved over time, which further contributes to the observed variation in epidemiology [3]. An illustrative instance of this disparity can be observed in the contrasting findings presented by the two most recent systematic reviews and meta-analyses on the subject. One systematic review and meta-analysis, which encompassed studies conducted globally between 2002 and 2018, reported a prevalence of approximately 14.50% (with a 95% confidence interval of 8.05% to 22.27%) of HDV co-infection among individuals carrying the hepatitis B surface antigen (HBsAg) and also living with HIV [21]. Conversely, another recent meta-analysis addressing the same topic suggested a lower prevalence of this ‘triple’ co-infection—HIV/HBV/HDV—reporting a pooled prevalence of 7.4% (with a 95% confidence interval ranging from 0.73% to 29.59%) [15]. These discrepant results highlight the need for up-to-date, region-specific data to inform targeted interventions.

The demographic makeup of our cohort further showcases the importance of understanding the specific transmission dynamics and risk factors prevalent in a regional context. It is worth noting the significant proportion of our cohort with a sexual mode of transmission (60%), and notably, 50% of the individuals self-identify as men who have sex with men (MSM). This demographic contrasts with recent data supporting increased HDV prevalence among intravenous drug users [13,20], a subgroup that is significantly underrepresented in our cohort (only 7%). Indicatively, data from the EuroSIDA and Swiss HIV Cohort Study showed that the prevalence of HDV co-infections was almost 6–15 times higher in IVDU than non-IVDU, i.e., 48.9 vs. 4.5% in Northwestern Europe, 55.6 vs. 3.7 in Eastern Europe and 50.9 vs. 8.1 in Southern Europe [13]. The high prevalence of MSM and the overall sexual mode of transmission in our cohort may suggest a distinct epidemiological pattern in the Greek PLWHIV population, predominantly driven by sexual transmission, as opposed to the hematogenous route more common among intravenous drug users.

Moreover, the clinical characteristics of our cohort, especially the high CD4 counts and the undetectable HIV viral loads, reflect effective HIV treatment, which may also influence the course and management of HBV and HDV co-infections. The interplay between effective antiretroviral therapy and reduced risk of viral infections and liver disease progression in co-infected individuals is an important aspect to consider. The observation of normal values in the liver enzymes within this subgroup may further signify the potential beneficial impact of effective HIV treatment on hepatic health. However, it is noteworthy that the single individual who tested positive for anti-HDV IgG had elevated liver enzymes (SGOT and SGPT both at 55 IU), indicating potential hepatic involvement. Despite this finding, no solid conclusions about the relative risk factors of HIV/HBV/HDV co-infection in Greece can be drawn from just one patient.

Most guidelines currently recommend testing for HDV in patients at high risk of acquiring the virus, including patients with HIV or HCV infection, men who have sex with men, commercial sex workers, IVDU, migrants from endemic countries without HBV vaccination programs, hemodialysis recipients, and patients with cirrhosis/HCC [7,22,23]. Recent EASL guidelines recommend screening for anti-HDV at least once in all patients with HBsAg (+), who should be re-tested according to clinical indications and yearly if increased risk of transmission persists [7]. However, despite the potential high disease burden and prevalence (4.0–18.8%), screening in HIV/HBV patients remains poor. The screening rate in the ICONA study was 78.7% [17], EUROSIDA and SHCS 56% [13] and barely reached 14% among the ATHENA cohort in the Netherlands [19]. A recent analysis comparing the rate of HDV diagnoses prior to and after the implementation of an anti-HDV reflex testing program for individuals positive for HBsAg in 1 academic (Hospital Universitario Valle d’Hebron, Spain) and 17 primary care settings showed that anti-HDV reflex testing in all the HBsAg-positive individuals led to a 5-fold increase in CHD diagnoses [24]. Prior to HDV reflex testing, a high proportion of HBsAg-positive individuals were not tested for HDV, particularly in primary care centers, while a considerable number of subjects (60%) had unknown risk factors, highlighting the importance of anti-HDV reflex testing and contrasting with the AASLD guidelines for anti-HDV testing only in those with risk factors [24].

Until recently, due to the lack of direct cytopathic effects by HDV, treatment protocols primarily aimed at curbing the necessary tools of the virus, shifting the focus to managing the coexisting HBV infection, for which numerous effective antiviral analogues are available [25]. Experience with IFN-α has been plentiful; however, the outcomes have been poor [7]. In July 2023, the European Medicines Agency (EMA) granted approval to bulevirtide, an innovative, first-in-its-class entry inhibitor, for the treatment of chronic HDV infection in adults with compensated liver disease, alone or in combination with an HBV-targeting analogue [7,26,27,28,29]. In line with clinical trial data, bulevirtide is effective and well-tolerated, with the HDV-RNA reduction following treatment in people living with HIV (PLWHIV) being similar to those without HIV [30]. Its administration was safe and well-tolerated, with no impact on the CD4 counts, HIV viral suppression, or HIV treatment regimen, while no major interactions with antiretroviral regimens—except for protease inhibitors—were observed [30,31].

At the moment, all patients with active liver disease, advanced liver fibrosis or compensated cirrhosis should be considered for treatment, as a successful treatment may result in improved long-term clinical outcomes [7]. Nonetheless, HBV monitoring, including the need to evaluate for HBV therapy, should continue while evaluating HDV, while baseline and ongoing imaging to screen for hepatocellular carcinoma should be performed every 6 months regardless of the anti-HDV therapy [7,26].

The observed low prevalence of HDV in our cohort may be interpreted as a reassuring sign. However, it simultaneously prompts considerations regarding the representativeness of our findings and the possibility of under-detection, particularly attributed to the small sample size and population diversity [32,33]. An intrinsic limitation of our study stems from the relatively modest prevalence of HBV/HIV co-infection within the national cohort from which our samples were drawn, with only 30 out of 696 individuals testing positive for HBsAg. Another limitation of our study is the potential non-inclusion of acute or window-period HBV/HDV infections due to our methodology of defining subgroups based on HBsAg positivity. This approach may not account for the initial phases of infection when HBsAg is not yet detectable, potentially leading to an underestimation of the actual prevalence of HBV/HDV co-infections. Additionally, while detailed data on HBV infection, vaccination rates, and past HBV infections among the cohort would provide valuable context, such information was not available in our dataset. Lastly, we are aware that a big proportion of difficult-to-treat populations, including IVDU and immigrant populations with poor access or engagement to care, might not be included in this study, hence representing an unknown pool of potentially increased seropositivity.

## 5. Conclusions

Our study highlights the low prevalence of anti-HDV IgG among PLWHIV in Greece, with less than 5% of the HBsAg-positive subgroup exhibiting positivity. Despite this low prevalence, the necessity for vigilant monitoring remains critical, especially considering the observed high rates of other sexually transmitted diseases. Emerging treatment options like bulevirtide offer hope for better management of HDV, reinforcing the necessity of not missing HDV cases in clinical practice.

## Figures and Tables

**Table 1:** Population characteristics.

Population Characteristics	HBsAg (+) (*n* = 30)
Demographics	
Age (IQR) (years)	54 (43–58)
Male sex (count) (%)	28 (93%)
Risk factors (count) (%)	
IVDU	2 (7%)
MSM	15 (50%)
Mode of transmission (count) (%)	
Bloodborne	1 (3%)
Sexual	18 (60%)
Unknown	11 (37%)
HIV stage	
A1–A3	22 (73%)
B1–B3	5 (17%)
C1–C3	3 (10%)
Treatment details (IQR)	
Years of treatment (years)	10 (6–15)
Viral load (copies/mL)	0 (0–0)
CD4 count (cells/µL)	738 (539–1006)
HIV treatment regimen (count) (%)	
NRTI (TAF/TDF + 3TC/FTC), yes (%)	27 (90%)
NNRTI (EFV, DOR), yes (%)	6 (21%)
INSTI (DTG, BIC, RAL), yes (%)	20 (71%)
PI (DRV/r), yes (%)	9 (30%)

Abbreviations: IQR—interquartile range; MSM—men who have sex with men; IVDU—intravenous drug use; NRTI—nucleoside reverse transcriptase inhibitor; TAF—tenofovir alafenamide; TDF—tenofovir disoproxil fumarate; 3TC—lamivudine; FTC—emtricitabine; NNRTI—non-nucleoside reverse transcriptase inhibitor; EFV—efavirenz; DOR—doravirine; INSTI—integrase strand transfer inhibitor; DTG—dolutegravir; BIC—bictegravir; RAL—raltegravir; PI—protease inhibitor; DRV/r—darunavir/ritonavir; HBsAg—hepatitis B surface antigen; HIV—human immunodeficiency virus; CD4—cluster of differentiation 4.

**Table 2:** Infectious disease panel and laboratory values.

Infectious Disease Panel (Count) (%)	HBsAg (+) (*n* = 30)
Anti-HDV IgG	1 (3%)
Anti-HDV IgM	0 (0%)
HCV IgG	2 (7%)
HAV IgG	16 (55%)
RPR/VDRL/TPHA (+)	9 (33%)
Other STD	1 (4%)
TB	1 (4%)
History of gonococcal disease	3 (11%)
History of chlamydia	2 (7%)
Laboratory Values (IQR)	
WBC (×10^3^/µL) [normal range: 4.0–11]	6.53 (5.12–8.48)
PLT (×10^3^/µL) [normal range: 150–400]	243 (204.75–264.25)
SGOT (U/L) [normal range: 5–40]	20 (17–26)
SGPT (U/L) [normal range: 5–40]	20 (16.25–30)
Alb (g/dL) [normal range: 3.5–5.5]	4.53 (4.36–4.73)
Ur (mg/dL) [normal range: 15–54]	30 (24–38)
Cr (mg/dL) [normal range: 0.9–1.6]	1 (0.85–1.1)

Abbreviations: IQR, interquartile range; HBsAg, hepatitis B surface antigen; HDV, hepatitis D; HCV, hepatitis C virus; HAV, hepatitis A virus; RPR, rapid plasma reagin; VDRL, venereal disease research laboratory; TPHA, treponema pallidum hemagglutination assay; TB, tuberculosis; HDV, hepatitis D virus; IgG, immunoglobulin G; IgM, immunoglobulin M; WBC, white blood cell; PLT, platelets; SGOT, serum glutamic-oxaloacetic transaminase; SGPT, serum glutamic-pyruvic transaminase; Alb, albumin; Glob, globulin; Ur, urea; Cr, creatinine.

## Data Availability

Data are available upon reasonable request to the corresponding author.
